# Supplementary material for: From CBC to clarity: Interpretable detection of beta-thalassemia carriers in imbalanced datasets
Source: PLoS One. 2025 Sep 18;20(9):e0331985. doi: 10.1371/journal.pone.0331985 (PMC12445568; doi:10.1371/journal.pone.0331985)
Supplement: Attachment — Submitted filename: Author feedback on reviewer-Thalassemia-PLOS.docx [file pone.0331985.s002.docx]

PONE-D-25-27555

Title: From CBC to Clarity: Interpretable Detection of Beta-Thalassemia Carriers in Imbalanced Datasets

PLOS ONE

Dear Editor,

I am very thankful for the comments on the review. According to them, we have made revisions and modifications to the paper. Please see the responses to review comments in the table below. The revised manuscript has also been submitted for consideration.

Our manuscript has been professionally proofread and edited.

Yours sincerely,

Dr. Nadia Sultan

Corresponding Author

**Reviewer #1:**

Thank you for your insightful suggestion regarding the ability of hematologists to identify β-thalassemia carriers using blood indices, and the particular challenge presented by borderline cases. To help you reconnect with your suggestions, we have reproduced your comments below, followed by our response.

| **Reviewer Comments-** | **Author Feedback** |
| --- | --- |
| The article entitled "From CBC to Clarity: Interpretable Detection of Beta Thalassemia Carriers in Imbalanced Datasets: explains using Machine learning approaches to detect carriers of B thalassemia. suggestion: hematologists could indicate the carriers by blood indexes, the difficulty is for boarder line indexes, if this method only detects these carriers please explain how it improves the detection rate. | We fully agree that experienced hematologists can often make accurate judgments based on established hematological indices. However, as you correctly noted, distinguishing β-thalassemia carriers becomes significantly more difficult in borderline or ambiguous cases—where index values fall near cutoff thresholds and visual inspection of CBC parameters alone may not offer conclusive classification.  In our study, this challenge has been addressed through following contributions:   1. **Improved Detection in Borderline Cases:** Our model does not rely solely on static thresholds (e.g., Mentzer Index ≤13), but also employs a Dominance-based Rough Set Approach (DRSA) with the VC-Domlem algorithm to generate interpretable decision rules. These rules capture hidden patterns across multiple CBC features and newly proposed indices (e.g., BTD), enabling detection even when individual parameters fall in borderline ranges. As detailed in Table VI of our manuscript, the model derives complex multi-attribute rules such as:   (MCHC ≥ 30) & (RDWCV ≥ 35) & (MI ≥ 26.7×10⁶) & (BTD ≥ 4.5×10⁹) ⇒ Carrier  Such combinations allow identification of carriers that may otherwise be misclassified using traditional univariate thresholds.   2. **Experimental Evidence of Improved Detection:** Our model was validated on an imbalanced real-world dataset, with 17% β-thalassemia carriers. Despite the class imbalance and borderline data exclusion during preprocessing, the DRSA model achieved a recall of 0.84 (on unseen data) which is representing meaningful improvements in carrier identification. Importantly, the DRSA maintained low false positive rates and high interpretability, enabling clinicians to trace and understand the reasoning behind predictions.   Although hematologists play a central role in diagnosis, our model augments clinical decision-making, particularly in ambiguous CBC profiles, by offering rule-based classification that enhances detection rates. |

**Reviewer # 2:**

Thank you for your positive comments and encouraging feedback on our manuscript. We are pleased to know that the originality of our approach—particularly the integration of the Dominance-based Rough Set Approach (DRSA) with the VC-Domlem algorithm—was appreciated, along with the clarity of writing and potential clinical impact.

We have carefully considered your valuable suggestions and incorporated in the manuscript. To help you reconnect with your suggestions, we have reproduced your comments below, followed by our response**.**

| **Reviewer Comments** | **Author Feedback** |
| --- | --- |
| This is a very interesting scientific study. The proposed approach is original, particularly in its use of the Dominance-based Rough Set Approach (DRSA) combined with the VC-Domlem algorithm to detect beta-thalassemia carriers using simple Complete Blood Count (CBC) data. The study is well-structured, methodologically sound, and shows strong potential for clinical application.  The manuscript is written in clear and well-structured English, and the figures are generally well presented. However, it would be helpful to include direct links to the cited references to facilitate access for readers. Additionally, the introduction should be made more concise.  I would also like to raise a question regarding the model's performance: the sensitivity for detecting carriers appears to be around 60%. What strategies do you propose to improve this sensitivity while maintaining the interpretability of the model? | In response to the suggestion, we have now included direct hyperlinks for all cited references in both the reference list and the EndNote file to improve accessibility for readers highlighted in the manuscript.  In light of your suggestion, we concise the introduction highlighted in the main body of manuscript:  1. **INTRODUCTION**  Beta-thalassemia (β-thalassemia) is a genetic disorder that may remain asymptomatic in many individuals, who unknowingly act as carriers and transmit the condition to future generations. This disorder is caused by mutations or alterations in the beta-globin gene (HBB), which is located on chromosome 11. Nearly 200 mutations have been identified as potential causes of β-thalassemia. While gene deletions can also lead to this disorder, they are relatively rare [1]. β-thalassemia presents a significant public health burden in various parts of the world. The global carrier frequency of β-thalassemia is approximately 1.5%, translating to approximately 80–90 million carriers worldwide [2]. Mediterranean regions such as Sardinia and Cyprus report notably high frequencies, approximately 12% to 15% [2]. Southeast Asia has one of the highest carrier frequencies, ranging from 5% to over 10% in various populations [2,3]. Each year, about 60,000 children are born with β-thalassemia major in developing countries [3]. Pakistan, with an estimated population of over 225 million, is believed to have around 10 million β-thalassemia carriers, representing 5% to 7% of the total population [4]. Approximately 5,000 children are diagnosed with β-thalassemia major annually in the country [4], placing a considerable burden on healthcare infrastructure. Early and accurate diagnosis could prevent many of these cases and save lives. Therefore, we focused on local data collection to accurately measure the danger of β-thalassemia carrier presence in terms of local demographics.  Since β-thalassemia carriers are generally asymptomatic, cost-effective screening is essential. A Complete Blood Count (CBC) test, which is rapid and inexpensive, is commonly used to detect carriers. Additional diagnostic tests—such as serum iron, HbA2 levels, ferritin, and iron-binding capacity—can also aid in diagnosis but are more expensive and not universally available in all healthcare settings.  The Mentzer Index (MI) is a very useful method for differentiating between iron deficiency anemia and β-thalassemia trait. The Mentzer Index is calculated by simply dividing Mean Corpuscular Volume (MCV) by Red Blood Cell Count (RBC). If the Mentzer Index is greater than 13, it means the patient has iron deficiency anemia and if the value is less than 13, it means that the patient has beta thalassemia trait [5]. This technique is particularly useful as a first-line screening method in areas where both iron deficiency and β-thalassemia are common [6]. Besides the Mentzer Index, several other hematological indices have been proposed to identify β-thalassemia carriers based on CBC results. When integrated with machine learning techniques, such data can significantly enhance the identification of thalassemia carriers, making it highly suitable for cost-effective screening in resource-constrained settings. When combined with machine learning methods, such data can aid in identifying thalassemia carriers therefore, making it highly suitable for cost-effective screening in resource-constrained settings [7].  In our current study, we applied the Dominance-based Rough Set Approach (DRSA) on a local dataset. DRSA is an extension of Classical Rough Set Theory (RST) that focuses on the dominance aspect of attributes and defines dominance relations [8]. We use the DRSA for calculating the reduced features, then used the Domlem algorithm (Variable Consistency Model) to generate decision rules. Domlem is a rule-induction algorithm that focuses on generating decision rules by considering the dominance relationships between different attributes[9-10]. These decision rules have been used to build a classifier for the dataset. The novelty of this research lies in the application of DRSA to an imbalanced medical dataset. Additionally, we introduced and evaluated a new index, Beta Thalassemia Detector (BTD), alongside traditional indices, to enhance predictive performance in this specific clinical context.  In the remaining paper, the following sections have been included: In Section 2, a brief definition of the basic concepts of DRSA and the Domlem algorithm. In Section 3, a review of related work is included. Research gaps are based on the literature review outlined in Section 4. In Section 5, details of the proposed methodology are presented. Model evaluation and results are discussed in Section 6. In Section 7, the conclusion and future work of this paper is given.  The concern regarding sensitivity (~60%) for carrier detection is well acknowledged. It is important to note that one of the major challenges in achieving higher sensitivity in our case stems from the **inherently imbalanced nature of the dataset**, where β-thalassemia carriers represent a small minority of the samples. In our work, we addressed this by using the Dominance-based Rough Set Approach (DRSA), which is well-suited for handling imbalanced and preference-ordered data. Still, to further improve sensitivity without compromising interpretability, we are exploring the following enhancements:   - **Feature Expansion with Derived Indices:** We are investigating the addition of more biologically meaningful derived indices beyond those currently used (e.g., refined BTD variants, ratios combining RDW, MCH, and platelet counts). These can be incorporated into the DRSA framework to yield more powerful but still interpretable rules. - **Multilevel Rule Sets:** Future work may include layered or hierarchical rule generation. For example, a primary DRSA rule set for general detection and a secondary “borderline detector” to capture harder cases—thus raising sensitivity through rule refinement. - **Incorporating Expert Knowledge:** Collaborating with hematologists to integrate domain knowledge into the preference ordering or dominance relations can help the algorithm generate rules that more closely align with subtle diagnostic cues, especially in borderline cases. - **Hybrid Models with Post-Hoc Interpretation:** Although not applied in this study, we are also exploring hybrid architectures where interpretable surrogates (like DRSA) are trained to mimic more sensitive black-box models, thus preserving clarity while enhancing performance.   We remain committed to the principle that clinical decision-support systems must be both accurate and explainable. Our future work aims to strike a better balance between these objectives—particularly by addressing the limitations imposed by data imbalance through thoughtful feature engineering, rule stratification, and expert-guided model enhancements. |
